# Association of American Medical Colleges

**Published:** 1876-12

**Authors:** 


### Editorial.

---

### ASSOCIATION OF AMERICAN MEDICAL COLLEGES.

The Provisional Association of American Medical Colleges, which was organized in Philadelphia last June, among other suggestions for the consideration of colleges, advised uniformity in fees. While an approach is attainable, and should be had, absolute uniformity in some instances is impracticable.

Medical colleges are the creatures of State governments, and cannot change their organic laws without the creator's consent. When, therefore, the charter of a college contains a provision on the subject of fees, neither the faculty nor board of trustees can change it. If a State law, whether incorporated in the charter or as a separate enactment, requires that a certain number of students shall receive a course of lectures each year free of charge, certainly the very existence of the institution depends upon compliance.

Uniformity in the conduct of medical colleges, as well as other institutions, professions and organizations, is certainly very desirable. In most of the essential particulars it can be observed in all of them. When, however, the fundamental principles of different organizations vary so as to preclude the possibility of uniformity on certain points, without changing the organic laws of their existence, great care should be exercised in the adoption of fraternal rules.

If, in consideration of a magnificent State endowment of a medical college, sufficient for ample remuneration to a faculty and for all incidental expenses, the charter should require that no fee at all should be demanded of students in attendance, hard worked and poorly paid teachers might envy, but they would have great difficulty in dislodging the lucky occupants, and in bringing the alumni of such an easy and necessarily prosperous institution into disrepute.

This, then, presents itself as a prominent difficulty in the way of establishing with all medical institutions the very desirable system of uniform college fees.

As friends of the proposition we desire to elicit an investigation of the subject; so that at the meeting in 1877 of the Permanent Association of American Medical Colleges, a feasible and practicable course may be suggested, and adopted by the body. While there are real difficulties in the way of making a satisfactory settlement of this question, we should not be influenced to abandon the cause, by unfounded arguments and imaginary evils. The analogy, for instance, that may seem to exist between medical college fees and private or public medical fees exacted from patients, is not real. The poor get sick and humanity demands the attention of the most convenient medical man, and when honesty and gratitude bring an offering of all the poor man has to spare, it should be received in liquidation of the debt, though it amounts to not more than half the usual fee. Not so with the medical student. Neither his life, nor health even, depends upon his being received as a beneficiary; for in this as in all other countries honorable pursuits invite on every side.

Alleged abuse of the beneficiary system seems to have given rise to discussion on this subject, and the example set in the Northwest of reducing fees—forcing itself gradually down to Georgia—calls for, at least, an attempt to harmonize on some uniform amount for a course. We hope something satisfactory is attainable. The Provisional Association gave expression on this subject as follows:

"*Resolved*, That anything like a wholesale system of such reduction or remission of established fees, or any open solicitation of recipients of such favors be regarded as in the highest degree improper, and that any college indulging in such practices deserves to forfeit its place on the *ad eundem* list of medical colleges."

Gradation of studies was considered in the Association, and a plan suggested which contemplates an extension of the term of study. Evidently the elementary or fundamental branches can be studied to greater advantage and with more permanent benefit to the student, without being interfered with by practical teaching; and we think a plan may be adopted for this purpose without compulsory extension of the term of study to three courses of lectures, as recommended. The first course may be made to include anatomy, physiology, materia medica and chemistry, and the second, all the practical branches. While the labors of teachers may be somewhat increased by this plan, the student will certainly profit greatly. This, with the separation of teaching from the licensing power, will insure the profession against poorly educated practitioners in future.

